# Antitumor Effects of a Novel Chromosome Region Maintenance 1 (CRM1) Inhibitor on Non-Small Cell Lung Cancer Cells In Vitro and in Mouse Tumor Xenografts

**DOI:** 10.1371/journal.pone.0089848

**Published:** 2014-03-04

**Authors:** Shuai Wang, Xiaohong Han, Jianfei Wang, Jiarui Yao, Yuankai Shi

**Affiliations:** Department of Medical Oncology, Cancer Institute/Hospital, Chinese Academy of Medical Sciences and Peking Union Medical College; Beijing Key Laboratory of Clinical Study on Anticancer Molecular Targeted Drugs, Beijing, China; Indiana University School of Medicine, United States of America

## Abstract

**Background:**

Chromosome Region Maintenance 1 (CRM1) is a nuclear exporter and its inhibitor has anti-tumor activity in various cancers. This study assessed the therapeutic efficiency of the novel CRM1 inhibitor KPT-185 on non-small cell lung cancer (NSCLC).

**Methods:**

NSCLC cell lines were treated with KPT-185 to assess changes in cell viability, cell cycle, apoptosis, and protein expression. NOD-SCID mice carrying NSCLC cell xenografts were orally treated with KPT-276, a clinical analog of KPT-185, to examine the efficacy and side effects of KPT-276 in vivo.

**Results:**

KPT-185 significantly reduced the viability of six NSCLC cell lines in a time- and dose-dependent manner, including epidermal growth factor receptor-tyrosine kinase inhibitor (EGFR-TKI)-resistant H1975 and H1650GR cell lines. In addition, KPT-185 induced these NSCLC cells to arrest at G1 phase of the cell cycle and caused apoptosis in a dose-dependent manner. KPT-185 treatment also reduced CRM1 protein levels in six NSCLC cell lines, and the reduction could be completely abolished by the proteasome inhibitor bortezomib. KPT-185 activated caspase 3, 8, and 9, but inhibited survivin expression in NSCLC cells. In a mouse H1975 cell xenograft model, tumor growth was significantly inhibited by oral KPT-276 administration, and there was no significant mouse body weight loss or other side effects.

**Conclusions:**

The current study demonstrated the anti-tumor effects of KPT-185 in NSCLC cells, including EGFR-TKI-resistant NSCLC cell lines. Further studies will assess anti-tumor activity of KPT-185 in a clinical trial for NSCLC patients.

## Introduction

Lung cancer is the leading cause of cancer death in the world, accounting for 1.3 million worldwide cancer-related deaths each year [1]. Histologically, approximately 85% of patients with lung cancers are non-small cell lung cancers (NSCLC) [2], most of which are diagnosed at an advanced stages of the disease and ineligible for curative surgery. Palliative treatment includes chemo- and radiotherapy and more recently, targeting therapy, such as epidermal growth factor receptor-tyrosine kinase inhibitors (EGFR-TKI) gefitinib, erlotinib, and icotinib. These therapies have improved the survival of patients with NSCLC [3]; however, patients who initially respond to EGFR-TKI treatments eventually develop acquired resistance. Thus, novel therapeutic agents with low toxicity and better outcomes are urgently needed for patients with NSCLC.

During human carcinogenesis or cancer progression, malignant cells acquire the ability to export key nuclear proteins that can influence treatment efficacy. These proteins include tumor suppressors and regulators of cell apoptosis, nuclear localization of which is required for their proper function [4]. Chromosome region maintenance 1 protein (CRM1 or called XPO1) is a member of the importin β superfamily of nuclear export receptors (karyopherins). Furthermore, CRM1 is the chief mediator of nuclear export, can interact with leucine-rich nuclear export signals (NESs), and transport proteins through nuclear pore complexes to the cytoplasm [5]–[7], including EGFR, p53 and nuclear factor of kappa light polypeptide gene enhancer in B-cells inhibitor, alpha (IκB-α) [8]–[10]. If the activity of CRM1-mediated export is blocked, protein function can be altered. Therefore, CRM1 inhibitors could be utilized as a novel class of targeting therapy against human cancer. Indeed, to date, many small molecule CRM1 inhibitors have been developed and with high anti-tumor activity, such as leptomycin B (LMB), ratjadone, goniothalamin, N-azolylacrylates, and CBS9106 [11]–[15]. These small molecule inhibitors covalently bind to the cysteine residue (Cys528) in the NES-binding groove of CRM1 protein [16]–[17]. A phase I clinical trial of LMB was conducted, but LMB was not recommended for further clinical development because of the high toxicity and lack of efficacy [18]. Thereafter, a number of LMB analogues have been reported with reduced toxicity [19].

More recently, another class of CRM1 inhibitor has been identified, including KPT-185 and KPT-276 (Karyopharm Therapeutics Inc.; Boston, MA, USA). These inhibitors are selectively inhibitors of nuclear export (SINE), and have been showed to be effective for treating certain types of cancers, including pancreatic cancer, acute myeloid leukemia, mantle cell lymphoma, resulting in significant growth inhibition and apoptosis of tumor cells without severe toxicity [20]–[22]. Meanwhile, the levels of CRM1 protein are elevated in lung cancer tissues when compared to normal lung tissues. Thus, in this study, we explored the therapeutic efficiency of these novel drug-like CRM1 inhibitors (i.e., KPT-185 and KPT-276) in NSCLC cells *in vitro* and *in vivo* to hopefully provide novel insight into these drugs for future target therapy of NSCLC.

## Materials and Methods

### Cell lines and reagents

The human NSCLC cell lines A549, H1650, H1975, H2228, and HCC827 were obtained from American Type Culture Collection (ATCC, Manassas, VA, USA). The H1650 Gefitinib-resistant (H1650GR) cell line was established in our laboratory by exposing the cell to increasing concentrations of gefitinib for 10 months. The resultant cell line H1650GR was resistant to gefitinib *in vitro* (IC_50_>30 µM). The NSCLC cell lines were cultured in RPMI 1640 medium, supplemented with 10% fetal bovine serum (FBS), 100 U/mL penicillin, and 100 µg/mL streptomycin (Invitrogen Life Technologies, Carlsbad, CA, USA).

KPT-185 and KPT-276 were provided by Karyopharm Therapeutics. Bortezomib was obtained from Selleck Chemicals LLC (Houston, TX, USA). The pan-caspase inhibitor Z-VAD-FMK was purchased from Calbiochem (San Diego, CA, USA). Antibodies against EGFR, GAPDH, cleaved-caspase-3, caspase-8, cleaved-caspase-9, poly- (ADP-ribose) polymerase (PARP), survivin, and secondary antibodies conjugated with horseradish peroxidase (HRP) against rabbit IgG and mouse IgG were obtained from Cell Signaling Technology (Danvers, MA, USA). Moreover, antibodies against CRM1, nuclear factor of kappa light polypeptide gene enhancer of B cells (NF-κB), and IκB-α were obtained from Santa Cruz Biotechnology (Santa Cruz, CA, USA). All other reagents and chemicals were obtained from Sigma-Aldrich (St. Louis, MO, USA).

### Cell viability assay

NSCLC cells were plated at a density of 6,000 cells per well in a 96-well plate and grown overnight. On the next day, the growth medium was replaced with fresh media containing serial dilutions of KPT-185 (up to 10 µM) or dimethyl sulfoxide (DMSO) as a control. In duplicate plates, NSCLC cells were exposed to KPT-185, gefitinib, or KPT-185 plus gefitinib. After the cells grew for up to three days, the cell viability was measured using a Cell Titer 96® Aqueous Non-Radioactive Cell Proliferation Assay (Promega, Fitchburg, WI, USA). The reagent was added to the cells and further incubated for 3 h. The absorbance was then measured at 490 nm with a micro-plate reader (Bio-rad, Hercules, CA, USA). The data were summarized as percentage of untreated (DMSO control) cells.

### Flow cytometric cell cycle and apoptosis assay

After treating NSCLC cells with KPT-185 for 48 h, they were stained with propidium iodide (PI) staining buffer (Cayman Chemical, Ann Arbor, MI, USA) for 30 min at room temperature and then measured by FACS cytometry (BD Biosciences, NJ, USA). The DNA histograms were analyzed using ModFit LT cell cycle analysis software (Verify Software House).

Cell apoptosis was detected with an Alexa Fluor 488 annexin V/Dead Cell Apoptosis Kit (Invitrogen) according to the kit instructions. Briefly, after treating with KPT-185 for 48 h, the cells from both suspension and adherence were collected and co-incubated with Annexin V-fluorescein isothiocynate (FITC) and PI, then measured by FACS cytometry (BD Biosciences). The percentage of Annexin V and PI negative cells was determined based on the dot plots of FITC and PI.

### RNA isolation and qRT-PCR

Total cellular RNA was isolated using the RNeasy Mini Kit (Qiagen, Hilden, NRW, Germany) and then reverse-transcribed into cDNA using the superscript III first-strand synthesis system for RT-PCR (Invitrogen) according to the manufacturer's instructions. The cDNA samples were further amplified by Real-time PCR with the following synthetic primers (Invitrogen): CRM1, 5′-GCCTCACTGAGATTGCTGGT-3′ and 5′-TGAAGGGCCTCCATAAGAGT-3′; and glyceraldehyde-3-phosphate dehydrogenase (GAPDH), 5′-GAAGGTGAAGGTCGGAGTC-3′ and 5′-GAAGATGGTGATGGGATTTC-3′. The PCR conditions were set in a 20 µL reaction mixture containing cDNA (2 µL), primers (400 nM each, 1 µL), and SYBR green master mix (2×, 10 µL) (Invitrogen). cDNA was amplified in a Lightcycler480 Real-time PCR system (Roche Applied Science, Penzberg, Germany) with the following cycles: 95°C for 10 min followed by 40 cycles of 95°C for 15 s and 60°C for 60 s. All samples were analyzed in triplicate and repeated three times. The relative expression levels of CRM1 were normalized to GAPDH using the 2-ΔΔCT method.

### Protein extraction and Western blot

After treating cells with KPT-185 for 48 h, the cells were washed twice with cold phosphate buffer saline (PBS) and lysed with the NE-PER buffer (Pierce Biotechnology, Waltham, MA, USA) according to the manufacturer's instructions. The supernatant (whole cell lysate) was quantified and then electrophoresed using sodium dodecyl sulfate polyacrylamide gel electrophoresis (SDS-PAGE). The proteins were then transferred onto a PVDF membrane (Millipore, Billerica, MA, USA) using electroblotting. After that, the membranes were blocked with 5% dried skimmed milk in Tris-buffered saline plus Tween 20(TBS-T) for 1 h at room temperature and then incubated with a primary antibody at 4°C overnight. On the next day, the membranes were washed with TBS-T thrice and further incubated with a secondary antibody-HRP conjugate at room temperature for 1 h. After washing with TBS-T again, the proteins bands were detected with Immobilon Western HRP detection substrate (Millipore). Positive images were recorded with a chemiluminescence instrument (ProteinSimple, Santa Clara, CA, USA).

### Immunofluorescence microscopy

The localization and expression of CRM1, EGFR, IκB-α, NF-κB and p53 were detected in the presence and absence of KPT-185 by immunofluorescence microscopy. NSCLC cells were treated with KPT-185 for 48 h and fixed for 30 min with 4% paraformaldehyde. Next, cell membranes were permeabilization by treatment with Triton X-100 (0.1% w/v) in PBS for 15 min. After blocking with blocking buffer composed of 5% normal goat serum for 1 h, cells were treated with primary antibody for 1 h. After washing with PBS, cells were treated for 1 h with a secondary FITC-conjugated antibody and DAPI. Photomicrographic images were recorded by using a fluorescence microscope (Olympus, Tokyo, Japan).

### Mouse NSCLC cell xenograft assay

Four-week-old female non-obese diabetic-severe combined immunodeficient (NOD-SCID) mice were purchased from the Institute of Zoology, Chinese Academy of Sciences, and subcutaneously inoculated in the flank region with a suspension of H1975 cells (5.0×10^6^ cells). Tumor volumes were measured thrice weekly and were calculated using the following formula: volume (mm^3^) = (width (mm))^2^×length (mm)/2. Once the tumor growth was stable (average size of 100 mm^3^), mice were randomly assigned into 3 groups and orally treated with vehicle (Pluronic F-68/PVP-K29/32), gefitinib (100 mg/kg qd for 3 weeks) or KPT-276 (100 mg/kg 3 times a week for 3 weeks) [22]. Growth curves of tumor cell xenografts were generated by plotting the mean relative tumor sizes +/− SE. Body weight changes (measured thrice weekly) were expressed as a ratio relative to the weight just before initial treatment. Mice were sacrificed when the one-dimensional tumor diameter reached 15 mm or following the loss of 10% of their body weight. The significance of these differences among different groups was evaluated using an ANOVA test. The protein levels of CRM1, EGFR and survivin in xenograft tumors were detected by immunohistochemistry. This study was approved by the Committee on the Ethics of Animal Experiments of Cancer Institute/Hospital, Chinese Academy of Medical Sciences.

## Results

### Inhibition of NSCLC cell viability by KPT-185

**Figure 1:**
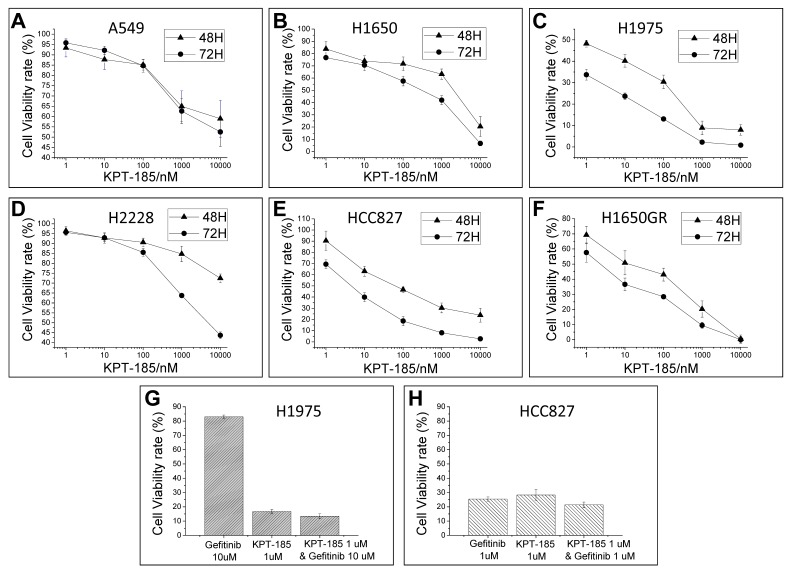
Effect of KPT185 on cell viability in NSCLC cells. Six NSCLC cell lines were treated with KPT-185 for 48 h or 72 h. Cell viability was suppressed by KPT-185 in a dose- and time-dependent manner (A∼F). KPT-185 inhibited cell viability in H1975 (G) and HCC827 (H) cells when compared to the effect of EGFR-TKI gefitinib treatment. (N = 3).

To assess the anti-tumor activity of the CRM1 inhibitor KPT-185, we performed cell viability assays in six human NSCLC cell lines. Cells were treated with serial dilutions of KPT-185 from 1 nM to 10 µM for 2 and 3 days. The data showed that KPT-185 reduced NSCLC cell viability in a dose- and time-dependent manner, with an IC_50_ from 1.3 nM to 46 µM (Figure 1A–F). In this study, each NSCLC cell line represented a different molecular subtype of NSCLC. Interestingly, KPT-185 equally inhibited the viability of EGFR-TKI-resistant H1975 and H1650GR cells and EGFR-TKI-sensitive HCC827 cells, indicating that KPT-185 may be a good candidate for the effective control of EGFR-TKI-resistant lung cancers. In addition, our data showed that there was no synergistic effect between KPT-185 and EGFR-TKI (gefitinib) treatment on tumor cell viability inhibition (Figure 1G & H).

### Induction of NSCLC cell cycle arrest by KPT-185

**Figure 2:**
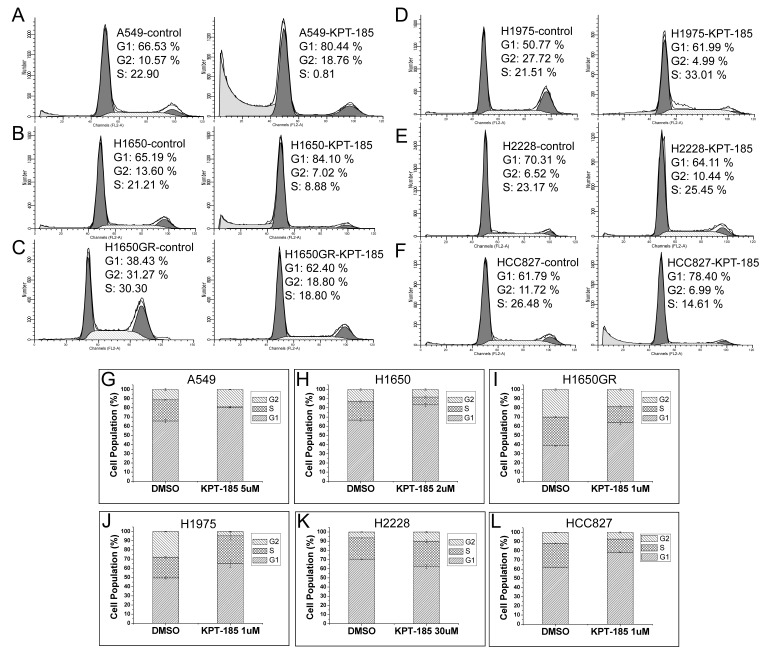
Effect of KPT-185 on the regulation of NSCLC cell cycle. Cells were arrested at the G1 phase of the cell cycle in KPT-185 sensitive cells. (N = 3).

To determine whether the reduction of cell viability following KPT-185 treatment was associated with changes in cell cycle distribution in NSCLC cells, we analyzed cell cycles in NSCLC cells after 24 h treatment with KPT-185. Compared to the control cells, KPT-185 treatment arrested NSCLC cells in the G1 phase of the cell cycle. However, this finding did not occur in H2228 cell which was not sensitive to KPT-185 treatment (Figure 2).

### Induction of NSCLC cell apoptosis by KPT-185

**Figure 3:**
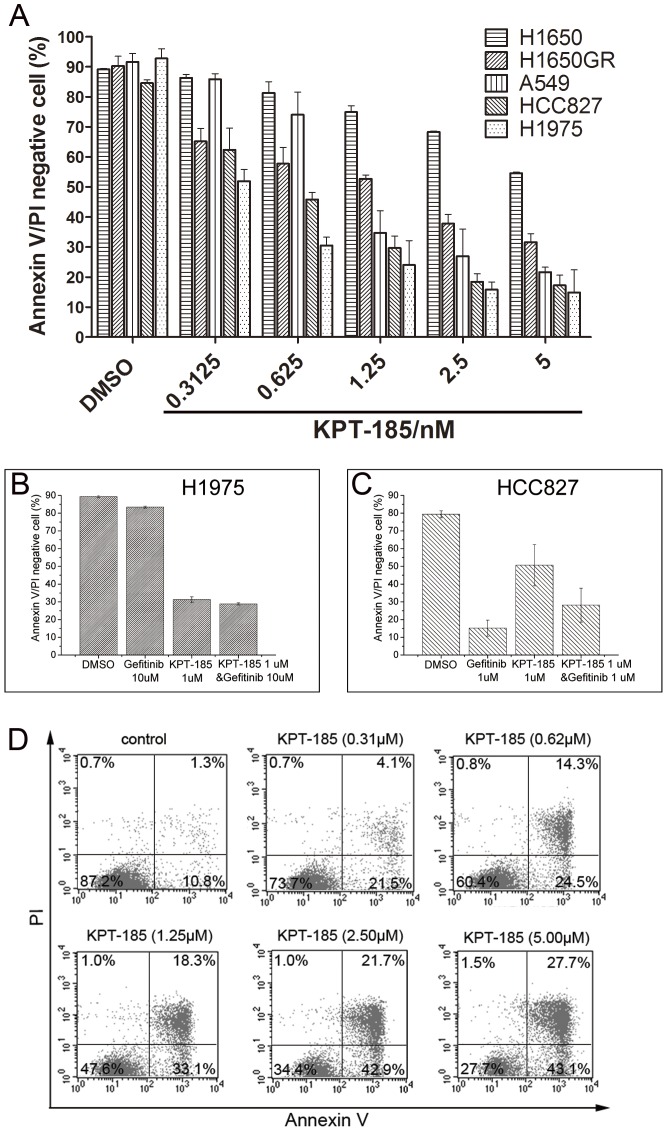
Effect of KPT-185 on apoptosis in NSCLC cells. The six NSCLC cell lines were treated with KPT-185 and then tumor cell apoptosis was assessed by flow cytometry assay. The data indicated a dose-dependent induction of cell apoptosis after 48 h of treatment (A). KPT-185 induced apoptosis in H1975 (B) and HCC827 (C) cells when compared with the EGFR-TKI gefitinib treatment. A representative data of apoptosis assay in H1975 cells was shown (D). (N = 3).

Since KPT-185 treatment induced NSCLC cell cycle arrest in the G1 phase of the cell cycle, we determined whether KPT-185 induced NSCLC cell apoptosis. Indeed, our data showed that KPT-185 treatment for 48 h increased the levels of Annexin V staining in a dose-dependent manner, indicating cell apoptosis (Figure 3A). Moreover, KPT-185 treatment induced H1975 cells to undergo apoptosis, but gefitinib did not have an effect. Meanwhile, both KPT-185 and gefitinib induced apoptosis in HCC827 cells (Figure 3B, 3C & 3D). In addition, treating cells with KPT-185 and gefitinib did not result in a synergistic effect on cell apoptosis.

### Downregulation of CRM1 and other proteins in NSCLC cells by KPT-185

**Figure 4:**
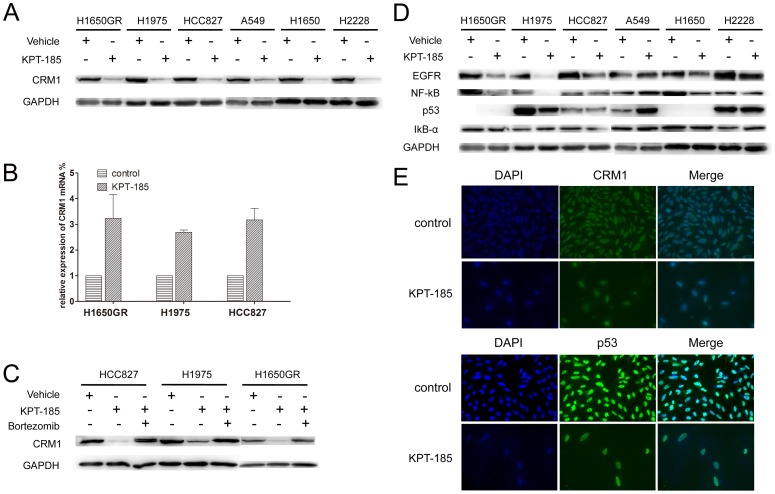
Effect of KPT-185 on the regulation of protein expression in NSCLC cells. The six NSCLC cell lines were treated with KPT-185 for 48 h. The expression of CRM1 protein was down-regulated (A & E), while the CRM1 mRNA levels were up-regulated (B, N = 3). In the presence of the proteasome inhibitor bortezomib, the reduction of CRM1 protein following KPT-185 treatment was almost blocked in H1975, HCC827, and H1650GR cells (C). EGFR expression was downregulated following KPT-185 treatment. The NF-κB and IκB-α proteins levels were not significantly affected. KPT-185 upregulated wild type p53 in A549 cells, downregulated mutant p53 in H1975 cell, and there was no effect in HCC827 and H2228 cell. In addition, p53 was not detected in H1650 and H1650GR cells (D&E, 400×).

Next, we analyzed the effect of KPT-185 on the regulation of CRM1expression in NSCLC cells. The level of CRM1 protein was markedly reduced after treatment with KPT-185 in all six NSCLC cell lines when compared with control cells (Figure 4A). However, the levels of CRM1 mRNA were higher in H1975, HCC827 and H1650GR cells after treatment with KPT-185 than that of the control cells (Figure 4B), indicating that reduction of CRM1 protein by KPT-185 was not at the transcriptional level. We then investigated whether KPT-185-reduced CRM1 protein expression was due to the activation of the ubiquitin/proteasome pathway. H1975, HCC827, and H1650GR cells were treated with KPT-185 in the presence or absence of bortezomib (10 nM; proteasome inhibitor) for 48 h. In the presence of bortezomib, the reduction of CRM1 protein by KPT-185 was almost blocked (Figure 4C), suggesting that KPT-185-induced CRM1 depletion requires activation of the ubiquitin/proteasome pathway. We then assessed the levels of various proteins that are shown to be exported by CRM1, such as EGFR, p53, and IκB-α. KPT-185 treatment also reduced EGFR expression in all six NSCLC cell lines (Figure 4D & Figure S1). However, there was no significant change in the levels of NF-κB and IκB-α in these cells, but IκB-α and NF-κB was accumulated in nucleus in KPT-185 treatment group when compared to the control group (Figure 4D, 4E & Figure S1). Interestingly, following treatment with KPT-185, the level of p53 was upregulated in A549 cells, which have a wild type p53, whereas p53 protein was downregulated and confined to nucleus in H1975 cells, which have a mutated p53 protein(Figure 4D & 4E). Meanwhile, there was no change in p53 protein levels in both the HCC827 and H2228 cells, and p53 protein was not detectable in H1650 and H1650GR cells (Figure 4D).

### Induction of caspase activation in NSCLC cells by KPT-185

**Figure 5:**
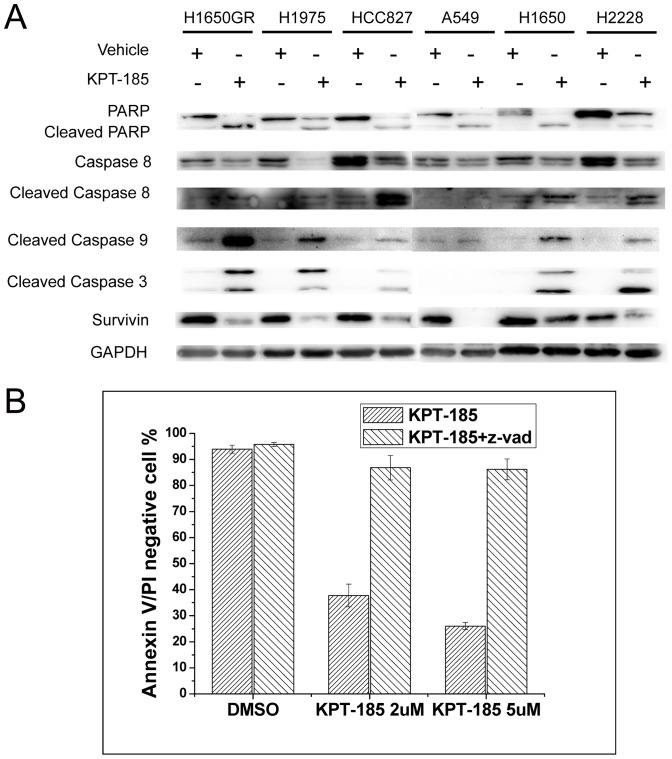
Effect of KPT-185 on the regulation of apoptosis related protein expression in NSCLC cells. The six NSCLC cell lines were treated with KPT-185 for 48 h. Caspases-8, -9 and -3 and PARP were activated or cleaved, but survivin was down-regulated (A). In the presence of the pan-caspase inhibitor Z-VAD-FMK, KPT-185-induced apoptosis was completely blocked in H1975 cells (B, N = 3).

To further elucidate the underlying molecular events by which KPT-185 induced apoptosis in NSCLC cells, we analyzed the activation and cleavage of caspases, PARP and survivin after treating the six NSCLC cell lines with KPT-185 for 48 h. Our data showed that when compared to control cells, caspases-8, -9 and -3, and PARP were activated or cleaved, whereas survivin, an inhibitor of apoptosis, was downregulated in NSCLC cells treated by KPT-185 (Figure 5A). We then treated H1975 cells with 100 mM of the pan-caspase inhibitor Z-VAD-FMK for 2 h before KPT-185 treatment. We found that H1975 cell apoptosis induced by KPT-185 was completely inhibited by Z-VAD-FMK (Figure 5B).

### Effect of KPT-276 on NSCLC cells in vivo

**Figure 6:**
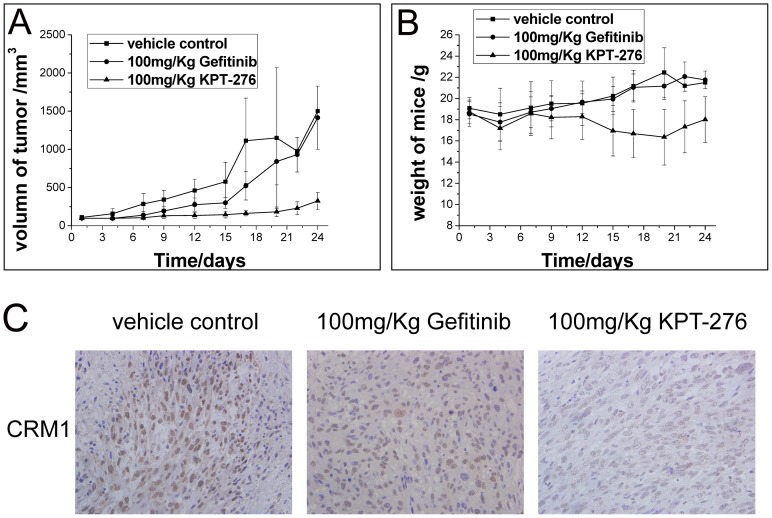
Effects of KPT-276 on mouse H1975 cell xenograft growth inhibition. NSCLC H1975 cells were transplanted into NOD-SCID mice. Mice with an established NSCLC cell xenograft were orally administered with vehicle, gefitinib, or KPT-276. Tumor cell xenograft growth was assessed for 30 days. Tumor growth curves showed a statistically significant suppression of H1975 cell growth in vivo when compared to vehicle or gefitinib treatment (*P*<0.05; A). Mice administered oral KPT-276 (100 mg/kg, three times a week for three weeks) tolerated the treatment well (B). The protein level of CRM1was detected in xenograft tumors by immunohistochemistry (C, 400×).

We then assessed the effects of the CRM1 inhibitor KPT-276 (the clinical equivalent to KPT-185) on NSCLC cells *in vivo*. We first transplanted NSCLC H1975 cells with an EGFR-TKI resistance mutation into NOD-SCID mice. After tumor xenografts were established, the mice were orally administered with vehicle, gefitinib, or KPT-276 treatments. We found that KPT-276 treatment significantly inhibited tumor growth when compare to the vehicle or gefitinib-treated tumor xenograft groups (*P*<0.05; Figure 6A). The mice tolerated the orally administered KPT-276 (100 mg/kg) treatments, as indicated by an average 6.64% weight loss (Figure 6B). In addition, chemotherapy-like side effects, such as diarrhea, were not observed. The protein levels of CRM1, EGFR and survivin in xenograft tumors were detected by immunohistochemistry. The expression of CRM1 was downregulated, and CRM1 was confined to nucleus in KPT-276 treatment group when compared to the control and gefitinib treatment group (Figure 6C). The expression of EGFR and survivin was downregulated in KPT-276 treatment group when compared to the control and gefitinib treatment group (Figure S2).

## Discussion

In this study, we explored the therapeutic efficiency of novel drug-like CRM1 inhibitors in NSCLC cells *in vitro* and *in vivo*. The results showed that the SINE CRM1 inhibitor KPT-185 reduced NSCLC cell viability and induced them to undergo apoptosis in a dose-dependent manner. KPT-185 also exhibited a strong anti-tumor activity in EGFR-TKI-resistant NSCLC cell lines. Moreover, KPT-185 also induced cell cycle arrest at the G1/S checkpoint in KPT-185-sensitive NSCLC cell lines, down-regulated levels of CRM1 and EGFR protein, accumulated p53, IκB-α and NF-κB in nucleus, and activated caspases-8, 3, and 9 proteins in NSCLC cells. Oral administration of KPT-276 significantly inhibited tumor xenograft growth in H1975-bearing NOD-SCID mice, which were resistant to EGFR-TKI treatment. Most importantly, the mice tolerated the KPT-276 treatment with minimal body weight loss and without severe toxicity. Further studies will assess the underlying molecular mechanisms of KPT-185 anti-tumor activity in a clinical trial for NSCLC patients.

Indeed, recent studies have demonstrated that CRM1 overexpression is associated with tumor progression and mortality in several human cancers [23]. For example, Zhang showed that CRM1 siRNA led to CRM1 protein knockdown and reduced mantle cell lymphoma (MCL) cell viability, and SINEs induced CRM1 protein translocation into the nucleus, where its expression was down-regulated [22]; thus, these data appeared to be opposite to former CRM1 inhibitors [13], [14], [24], [25]. In the current study, we demonstrated that KPT-185 reduced NSCLC cell viability, induced apoptosis, and arrested NSCLC cells at the G1 phase of the cell cycle. These data are consistent with previous studies on CRM1 inhibitors in different human cancers [13], [14], [22]–[25]. Our current data further showed that the SINE inhibitor KPT-185 down-regulated CRM1 protein levels in NSCLC cells through KPT-185-induced proteasome degradation of CRM1 protein. Thus, one possible cytotoxic mechanism of CRM1 inhibitors is via the ubiquitin/proteasome pathway, reducing the export of tumor suppressor proteins (TSP) from the nucleus to the cytoplasm, such as p53, IκB-α and NF-κB. Etchin et al. further demonstrated that CRM1 inhibitors could bind to the groove of CRM1 protein that was usually occupied by the NES, and block CRM1-directed protein export [21].

A number of studies have demonstrated that NF-κB activation is essential to maintain tumor cell viability, and inhibition of NF-κB activity alone is sufficient to induce cell death [26], [27]. However, in our current study, we did not observe any significant changes in NF-κB and IκB-α expression levels in NSCLC cells after KPT-185 treatment, but IκB-α and NF-κB was partially accumulated in nucleus in KPT-185 treatment group. CRM1 is known to export wild type p53 from the nucleus into the cytoplasm of cancer cells via its C-terminal NES, allowing for the efficient degradation of p53 by proteasomes. In our current study, we found that inhibition of CRM1 activity by KPT-185 prevented p53 from exiting the nucleus, resulting in nuclear p53 accumulation and stabilization in p53 wide type NSCLC A549 cells. In H1975 cells, which have a mutant p53, KPT-185 treatment down-regulated p53 levels and confined p53 to nucleus. In addition, p53 levels were stable in both HCC827 and H2228 cells, suggesting that apoptosis in these cells is p53-independent. Surprisingly, p53 was not detectable in H1650 and H1650GR cells. The H1650 cell line has been previously described as a cell line carrying mutant type of p53 [28].

EGFR regulates important tumorigenic processes, including proliferation, apoptosis resistance, angiogenesis and invasion, and is frequently overexpressed during the development and progression of NSCLC [29]. EGFR can be detected in the nucleus and exported to the cytoplasm by CRM1. In our study, we demonstrated that EGFR expression was reduced following KPT-185-reduced CRM1 expression in NSCLC cells. Noske et al. [30] showed the inverse association of EGFR with CRM1 expression in ovarian cancer tissues. Levels of EGFR protein expression were reduced after exposure of ovarian cancer cells to leptomycin B (LMB), suggesting that CRM1 plays a role in the intracellular transactivation of EGFR [30]. In contrast, Lo et al. detected increased nuclear EGFR levels following LMB treatment in human epidermoid carcinoma A431 cells [31], indicating that increased nuclear EGFR by LMB provides a plausible mechanism by which cells shuttle cell-surface EGFR through the nuclear pore complex and into the nuclear compartment.

Survivin is a member of the inhibitors of apoptosis family and protects against apoptosis by either directly or indirectly inhibiting the activation of caspases [32]. PARP is a substrate of caspase and its activation leads to apoptosis. In our current study, KPT-185 was able to activate caspases-8, -9 and -3, PARP, but inhibit survivin in all six NSCLC cell lines. Our current data also demonstrated that KPT-185 was able to induce apoptosis in both EGFR-TKI-sensitive and -resistant NSCLC cells. Furthermore, we found that the pan-caspase inhibitor Z-VAD-FMK completely prevented KPT-185-induced apoptosis in H1975 cells. Our results showed that SINE-induced apoptosis was dependent on caspase activation in NSCLC cell lines.

KPT-185 has been shown to possess unsuitable pharmacokinetic properties *in vivo*, either subcutaneously or orally. However, KPT-276, structurally related to KPT-185, is suitable for oral therapy [22]. Mutka et al. noted that LMB has off-target effects against proteins other than CRM1 and that these off-target effects might contribute to LMB's side effects (∼20% body weight loss) [33]. In our current study, we utilized KPT-276 at a dose of 100 mg/kg three times per week for three weeks to treat mice carrying NSCLC tumor cell xenografts. We found that these mice tolerated (less than 10% body weight loss) the treatment. In addition, KPT-276 exhibited high anti-tumor activity in EGFR-TKI-resistant NSCLC cell xenografts. These results indicate that KPT-276 may be a useful CRM1 inhibitor for clinical treatment of NSCLC patients, especially for EGFR-TKI-resistant patients.

## Supporting Information

Figure S1 **The localization and expression of EGFR, IκB-α, and NF-κB were detected in the presence and absence of KPT-185 by immunofluorescence microscopy.** The expression of EGFR was downregulated, and IκB-α and NF-κB was accumulated in nucleus in KPT-185 treatment group when compared to the control group (400×).
(TIFF)

Figure S2 **The protein levels of EGFR and survivin were detected in xenograft tumors by immunohistochemistry.** The expression of EGFR and survivin was downregulated in KPT-276 treatment group when compared to the control and gefitinib treatment group (400×).
(TIF)
