# Supplementary figures and images for: Antitumor Effects of a Novel Chromosome Region Maintenance 1 (CRM1) Inhibitor on Non-Small Cell Lung Cancer Cells In Vitro and in Mouse Tumor Xenografts
Source: PLoS One. 2014 Mar 4;9(3):e89848. doi: 10.1371/journal.pone.0089848 (PMC3942386; doi:10.1371/journal.pone.0089848)

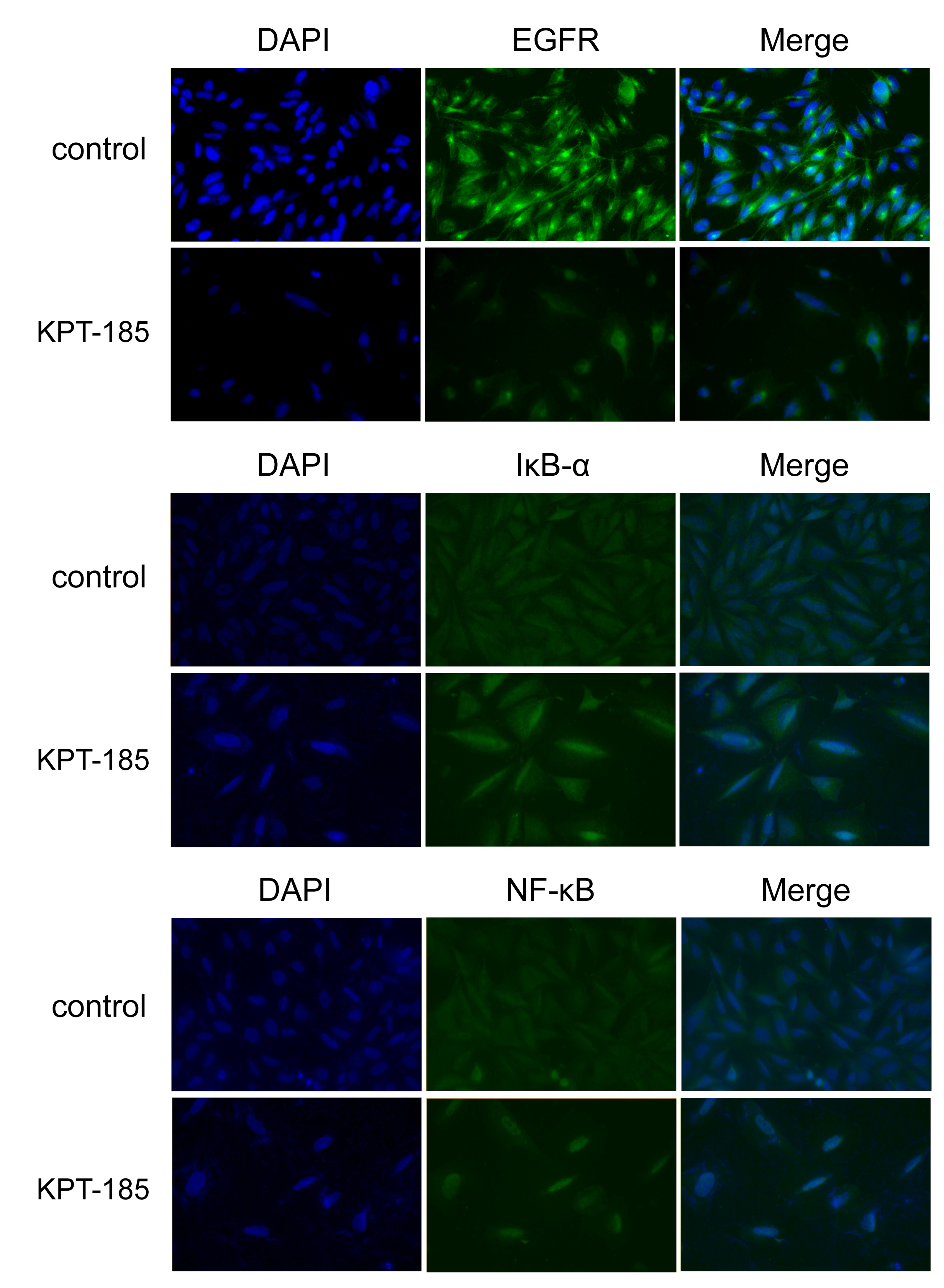

Supplement: Figure S1 — The localization and expression of EGFR, IκB-α, and NF-κB were detected in the presence and absence of KPT-185 by immunofluorescence microscopy. The expression of EGFR was downregulated, and IκB-α and NF-κB was accumulated in nucleus in KPT-185 treatment group when compared to the control group (400×). (TIFF) [file pone.0089848.s001.tiff]

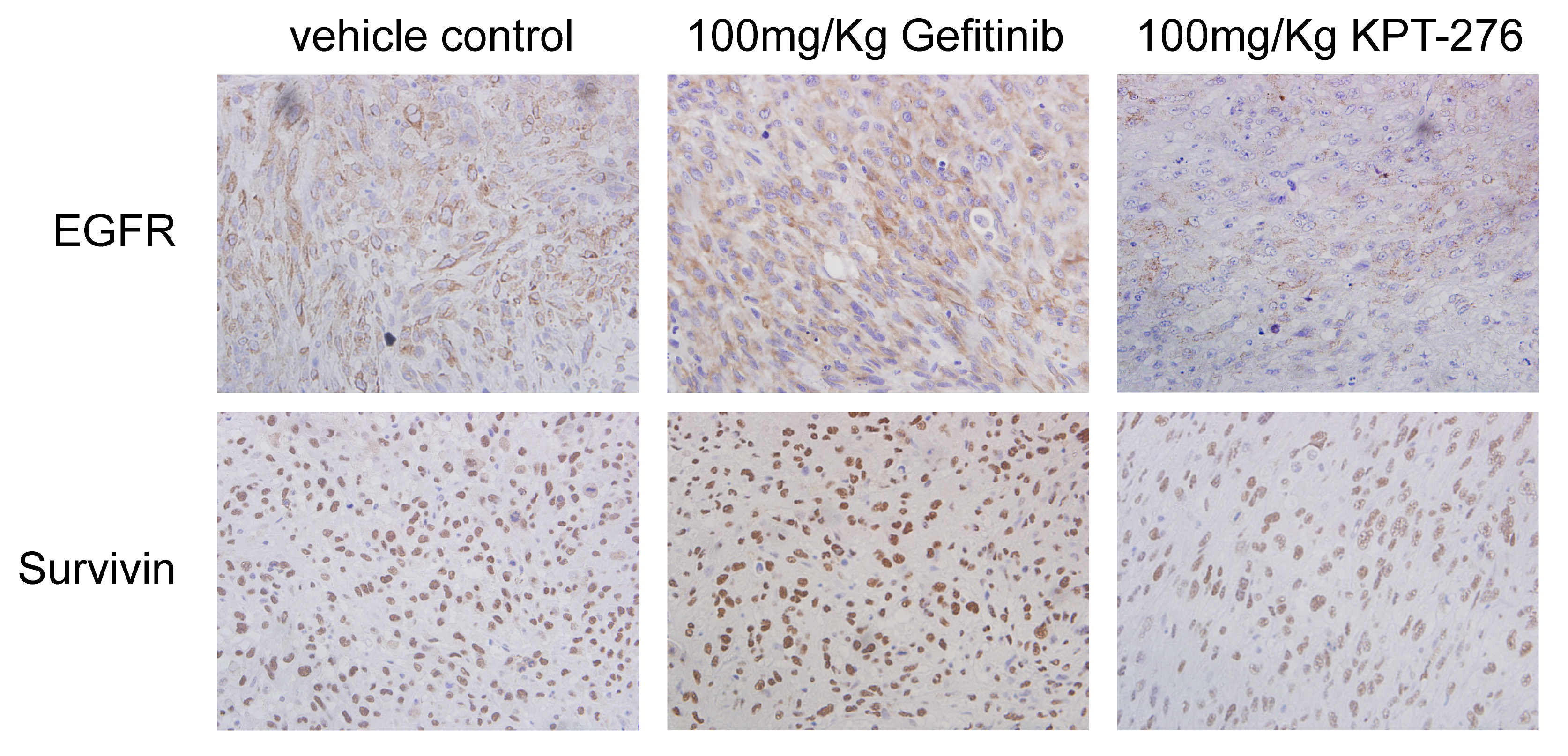

Supplement: Figure S2 — The protein levels of EGFR and survivin were detected in xenograft tumors by immunohistochemistry. The expression of EGFR and survivin was downregulated in KPT-276 treatment group when compared to the control and gefitinib treatment group (400×). (TIF) [file pone.0089848.s002.tif]
